# Use of contraceptives and associated factors among male adolescents in rural secondary schools, Coast Region, Tanzania: a school-based cross-sectional study

**DOI:** 10.1186/s40834-024-00268-w

**Published:** 2024-03-01

**Authors:** Ally Abdul Lyimo, Jia Guo, Stella Emmanuel Mushy, Beatrice Erastus Mwilike

**Affiliations:** 1 Department of Community Health Nursing, Muhimbili University of Health and Allied Sciences, Dar Es Salaam, Tanzania; 2 Department of Nursing, The Third Xiangya Hospital, Central South University, Changsha, 410013 China; 3 Department of Community Health Nursing, Muhimbili University of Health and Allied Sciences, Dar Es Salaam, Tanzania

**Keywords:** Contraceptive, Family planning, Knowledge, Male Adolescents, Use

## Abstract

**Background:**

Teenage pregnancy is still one of the reproductive health concerns facing adolescents in Tanzania. The problem has been associated with physiological, psychological, and social changes and increases the risk of unsafe abortion and adverse maternal, fetal, and neonatal outcomes among adolescents. Low utilization of contraceptive methods among adolescents is one of the key causes. The strategy of involving male adolescents in sexual and reproductive health programs can increase the rate of contraceptive use among adolescents, thereby preventing teenage pregnancy.

**Objective:**

To examine factors associated with the uptake of contraceptives among male secondary school adolescent students.

**Methods:**

This was a cross-sectional study conducted in rural secondary schools in Kisarawe District, Coast Region, Tanzania. Multi-stage sampling methods were used to recruit participants. Descriptive and multiple regression analyses were conducted to assess the prevalence and factors associated with contraceptive use. 95% confidence interval and *p*-value < 0.05 were considered statistical significance. Univariate and multivariate logistic regression were tested for the Crude Odds Ratio (COR) and Adjusted Odds Ratio (AOR) respectively.

**Results:**

The study involved 422 male students with the majority of them 58.1% aged 17–19 years, 50.2% were Muslim, 76.3% were studying in government schools, 62.3% were from households size of 4–6 members, 87.4% were not in a relationship, and 64.2% were living with both parents. Less than half (38.9%) of male students reported ever having sex in their lifetime, and among them, very few (29.8%) used any method of contraceptive. The reported lowest age for the first sex was 10 years. The male condom was the most method used (69.4%) and Pharmacy/Chemist Shops were the common source of contraceptive services (55.1%). Students who had adequate knowledge of contraceptives were more likely to report the use of contraceptive methods compared to those who had inadequate knowledge (AOR = 2.704, 95% CI: 1.220–5.995, *p* = 0.014). Participants in Private schools were 4.3 times more likely to report the use of contraceptives than those in government schools (AOR = 4.347, 95% CI: 1.758–10.762, *p* = 0.01). Students in a relationship were 3.5 times more likely than those not in a relationship to report the use of a contraceptive method (AOR = 3.51, 95% CI: 1.421–8.670, *p* = 0.006).

**Conclusion:**

The study found the low use of contraceptives among male adolescents who ever had sex in their lifetime. Thus, it’s suggested that age-tailored comprehensive sexual and reproductive health education should start to be taught from a very young age as adolescents initiate sex at an early age. Also, Teenage pregnancy prevention programs should involve males as the key players during the development and implementation of the program as most of the decisions among partners are from men.

## Introduction

Teenage pregnancy is a global reproductive health concern and it is approximated that each year, twenty-one million teenage girls become pregnant in developing countries [1]. This problem has been facing secondary school students in Tanzanian for decades, as many of them engage in sex in a young age [2]. A 2022 Tanzania Demographic and Health Survey and Malaria Indicator Survey (TDHS) reported that 22% of girls aged 15–19 years old have been pregnant between 2017–2022 and most of them (24.4%) are from rural areas [3].

Adolescent pregnancy is related to physiological, psychological, and social changes, and increases the risk of unsafe abortion, adverse maternal, fetal, and neonatal outcomes, HIV, and other sexually transmitted infections (STIs) [4, 5]. Teen mothers aged 10–19 years are at higher risk of suffering from eclampsia, puerperal, endometritis, and systemic infections than women aged 20 years old and above, and their babies are prone to low birth weight, preterm birth, and severe neonatal conditions [1]. The problem also could have an impact such as a lack of education and vocational opportunities as pregnant girls are forced to drop out of school and it is unlikely that they will return to education at a later stage [6–8].

International evidence recommends the effective utilization of contraception as a promising strategy to prevent adolescent pregnancy, ensure their health and development, and improve their opportunity for education and reproductive livelihoods [9, 10]. Effective use of modern contraceptives among adolescents would reduce unintended pregnancies by 6 million annually, hence averting about 2.1 million unplanned births, 3.2 million abortions, and 5600 maternal deaths [11]. Many interventions are available to deal with Sexual and Reproductive challenges among adolescents. However, the rate of utilization of contraceptives among adolescents globally is still low, over 20 million adolescents who require contraceptive services are not using any of the methods [12, 13]. Only 9.8% and 5.1% of adolescents were reportedly using any modern methods of contraception and traditional methods respectively in twelve countries of low- and middle-income [14]. In Tanzania, according to the 2022 Demographic and Health Survey and Malaria Indicator Survey report, only 18.4% and 3.2% of adolescents aged 15–19 used any method of modern contraceptive and traditional contraceptive respectively [3].

Male involvement in the prevention of teenage pregnancy through increasing the utilization of contraceptives is the current targeted strategy worldwide as the majority of programs focus chiefly on young women [15, 16]. A well-informed male adolescent about contraception may influence whether he will use and choose what method of contraception. Also in a situation where women have less freedom over their reproductive health, it may influence general contraceptive use [11]. There are several contraceptive methods such as condoms, vasectomy, withdrawal, and the Standard Days Method which require direct or indirect participation of men [17]. Despite all efforts to extend the role of the male in sexual and reproductive health, young men are still not as much involved as females in playing a critical role [18].

Tanzania is in the process of adopting the strategy to involve males in sexual and reproductive health programs, and some of the implemented programs focused on out-school males hence very little is known about the involvement of in-school male adolescents in the prevention of teenage pregnancy [2, 19, 20]. Therefore this study aimed to examine the uptake of contraceptives among secondary school male adolescents their knowledge, and the associated factors in a rural Cost region of Tanzania.

## Study methodology

### Study design and setting

A quantitative school-based cross-sectional study was conducted among secondary schools located in a rural area of Kisarawe District, Cost Region-Tanzania, from October 2020 to November 2020. Cost Region is among the regions with a high percentage of teenage childbirth (19.8%) with a national average of 22% and a high percentage of women aged 15–19 with unmet needs (13.9%) and demand (65.8%) for family planning [3].

### Eligibility criteria

The study was conducted among male students aged 11–19 years old attending secondary schools in a rural area of Kisarawe District, Cost Region-Tanzania as it has been reported that the youngest age at which adolescents become sexually active in Tanzania is 11 years old [21]. Male students with hearing impairment, loss of vision, sick, and not mentally fit were excluded from participation in the study.

### Sample size calculation

The sample size of the study was 422 participants. The sample size was estimated using the Cochrane formula [22]. As no study reported the proportion of use of contraceptives among male adolescents in Tanzania, the proportion was assumed to be 50%. Assumptions of 5% maximum acceptable error and 95% confidence interval with an allowance of 10% for the possible non-response were used for the calculation of the sample size.

### Sampling method

A multi-stage cluster-sampling method was used to recruit a total of 422 participants. All secondary schools in a rural area of Kisarawe District formed the primary sampling frame. Six secondary schools (Four from Government schools and two from private schools) were randomly selected from the primary sampling frame using the lottery method. Every stream in a selected school formed a secondary sampling frame, whereby students were enrolled using a systematic random method. To obtain a total number of students per school, the total study sample size (422) where divided by the number of selected schools (6). To determine the sample size required per class/stream, the total number of participants in each school was divided by the number of classes/streams in that specific school. The sampling interval (n) was obtained by dividing the number of students in each class/stream by the required sample size of that class/stream. The first participant was selected randomly by lottery method and other students were recruited at a regular interval (n) from the secondary frame.

### Research instruments and definition of variables

A validated semi-structured questionnaire in the Swahili language adapted from authors Dangat, C.M., and B. Njau, with an internal reliability coefficient of 0.71 was used [23]. The permission to adopt the tool was sought through the author’s email. The questionnaire had four parts whereby section one was used to collect socio-demographic data, section two collected information on contraceptive knowledge, section three collected data regarding the perception towards the use of contraceptive methods, and section four was used to gather information on the use of contraceptive methods. The tool was modified to meet the study objectives, and reviewed by research experts for content validation, and the internal reliability determined by Cronbach’s alpha was 0.740.

### Study variables

#### Independent variables

Socio-demographic characteristics included Age; (Categorized as 13–16 years and 17–19 years), Study Year; (Categorised into six classes Form I, Form II, Form III, Form IV, Form V, and Form VI). Religion; (Categorised as Muslim and Christian), Type of school; (Categorized as Government and Private school). Household Size; (Categorized into three groups, 1–3, 4–6, and 7 and above), Relationship status; (Categorized as Yes and No), and Staying with parents; (Options were “Yes” with both parents, “Yes”, with single parents and “No”).

Contraceptive knowledge was also identified as an independent variable. This was defined as the participant’s ability to respond correctly to questions regarding contraceptive methods and their functions. The Section had 11 items and each correct response was scored 1. The knowledge was categorized into Inadequate Knowledge and Adequate Knowledge, where a total score of 0–3 and 4–11 was classified as Inadequate Knowledge and Adequate Knowledge respectively [23, 24].

#### Dependent variables

The main outcome variable of the study was the use of the contraceptive method. This was a dichotomous variable defined as self-reported use of any form of contraceptive method among male students who had sex in a lifetime. Participants were asked to say “yes” or “no” in response to the question regarding contraceptive use.

### Data collection procedure

Data were collected after receiving an ethical clearance letter and permission to conduct the study in the Kisarawe district. Research assistants were trained on the purpose of the study and the use of the data collection tool before the process of data collection. Since the topic under study was very sensitive. We visited the selected schools and made an appointment with students. Data were collected individually without the presence of other students in an empty and quiet room to ensure confidentiality and work trust. Informed consent was obtained before distributing a self-administered semi-structured questionnaire. Each participant spent a minimum of 20 min to fill the questionnaire. Code numbers were used instead of names to ensure confidentiality and after completing filling out the questionnaire, the research assistants collected the questionnaires and sealed them in an envelope.

### Data management and analysis

Data coding, checking, and cleaning were done daily during the data collection process for validation and to ensure consistency. Frequencies, percentages, and mean scores were computed to describe socio-demographic data, knowledge, and use of contraceptives in the study population. Pearson’s chi-square and odds ratio were used to test the association between explanatory variables (socio-demographic characteristics) and outcome variables (Knowledge of contraceptives, and contraceptive use). The criterion of 95% confidence interval and *p*-value < 0.05 were considered as statistical significance. Multivariate logistic regression model analysis was conducted to explore the predictors of contraceptive use. All predictor variables that were statistically associated with contraceptive use using Chi-square were included in the multiple logistic regression model analysis. Univariate logistic regression analysis was conducted first. Each predictor variable was tested individually with the main outcome variable to give the Crude Odds Ratio (COR). Then multivariate logistic regression analysis was conducted in which predictor variables were combined in a test to provide the Adjusted Odds Ratio (AOR). Analysis was conducted using the Statistical Package of Social Sciences (SPSS) computer program version 25.

## Results

### Socio-demographic characteristics of male adolescents

A total of 422 male students were approached for participation in the study, of which a response rate was 100.0%. The mean age of the respondents was 16.8 years old (standard deviation (SD) = 1.5) with the majority (58.1%) aged 17–19 years. More than half of the respondents 50.2% were Muslim. The majority 76.3% were from government schools and the remaining 23.7% were from private schools. Most of the male adolescents 62.3%( *n* = 263) were staying from homes with a total of 4–6 family members. 87.4% (*n* = 369) of the students were not in a relationship during the time of the survey, and 64.2% (*n* = 271) were living with both parents (Table 1).

**Table 1:** Socio-demographic characteristics of the respondents (*n* = 422)

Variable	Frequency (n)	Percent (%)
**Age Group**		
13–16	177	41.9
17–19	245	58.1
**Age at first sex(*n* = 164)**		
10–13	45	27.4
14–16	90	54.9
17–19	29	17.7
**Study Year**		
Form I	70	16.6
Form II	70	16.6
Form III	70	16.6
Form IV	71	16.8
Form V	70	16.6
Form VI	71	16.8
**Religion**		
Muslim	212	50.2
Christian	210	49.8
**Type of School**		
Government School	322	76.3
Private School	100	23.7
**Number of Family members**		
1–3	29	6.9
4–6	263	62.3
7 and above	130	30.8
**Are you in a relationship?**		
Yes	53	12.6
No	369	87.4
**Stay with parents**		
Yes with Both parents	271	64.2
Yes with Single parent	116	27.5
No	35	8.3

*Abbreviations*: *n* number participants, *%* percent of participants, *p* Statistical significance at < 0.05 level

### Knowledge of contraceptives among male adolescents

**Fig. 1:**
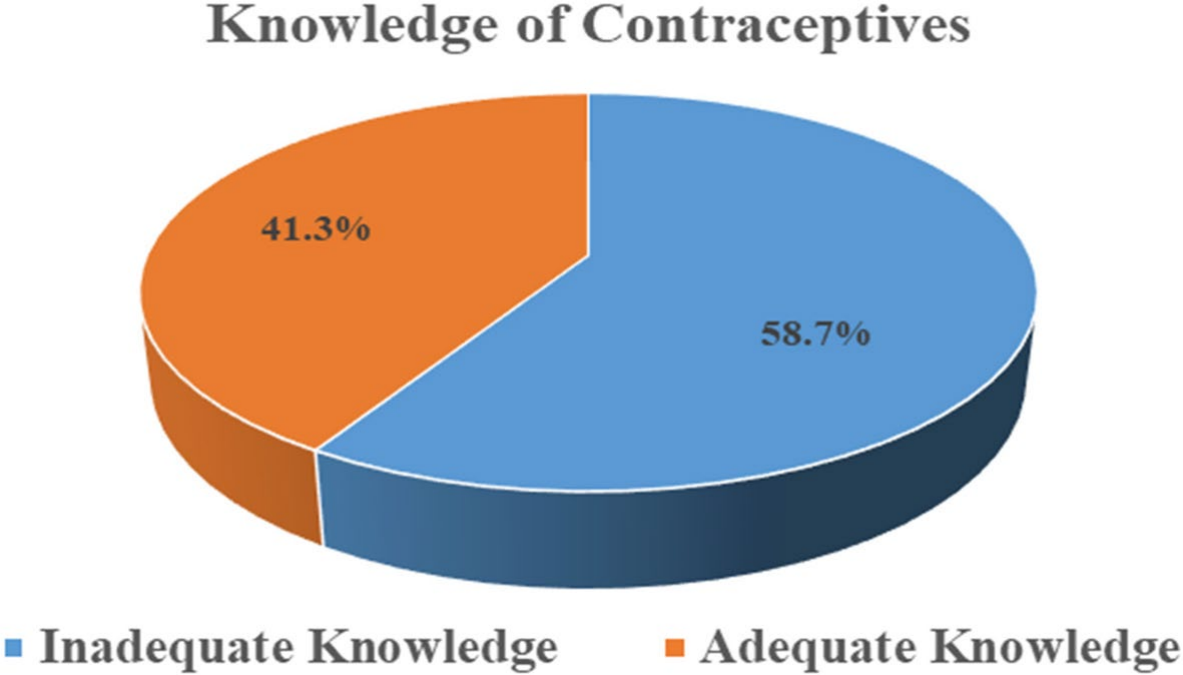
Level of contraceptive knowledge

**Fig. 2:**
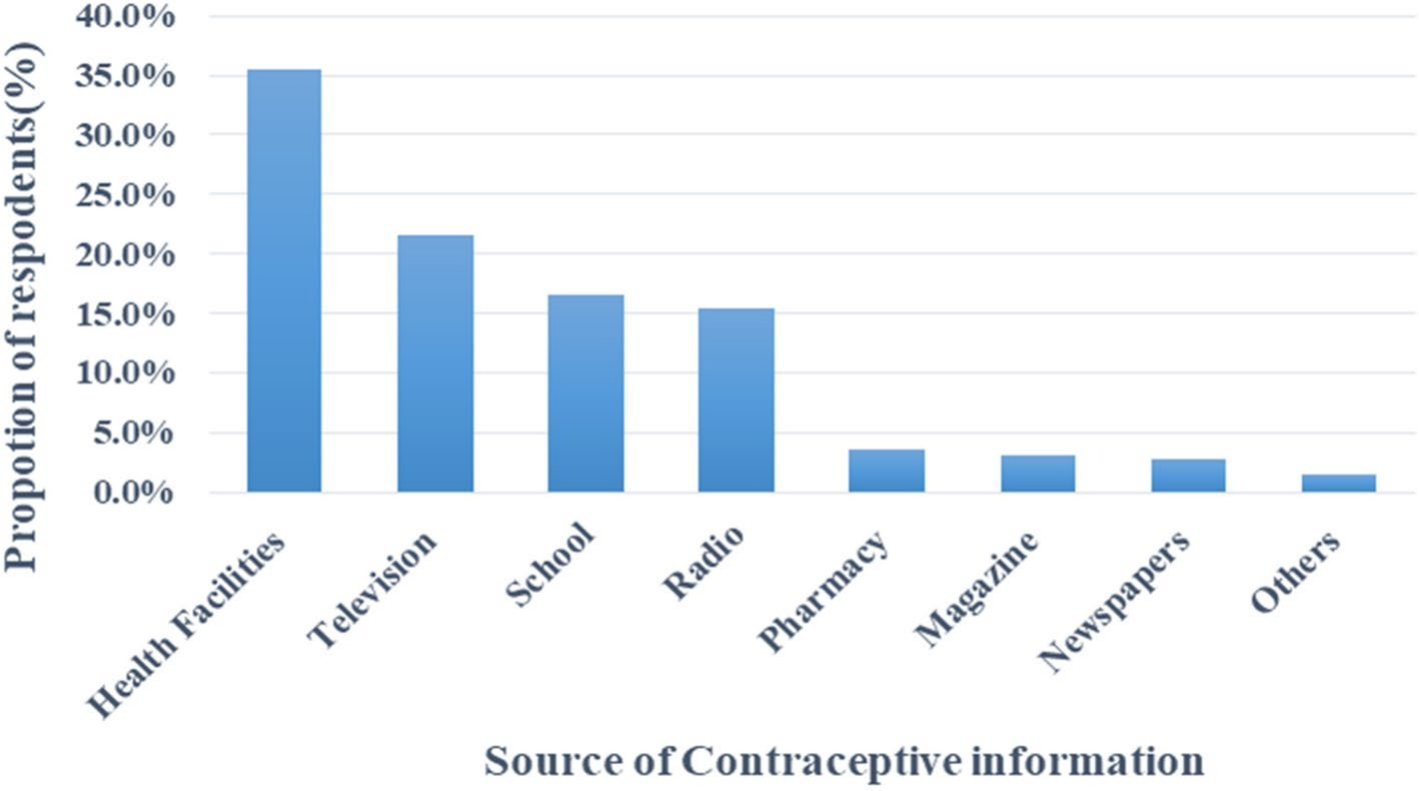
Sources of contraceptive information

More than half (58.7%) of participants had inadequate knowledge of contraceptives (Fig. 1). Male condom was the most common contraceptive method mentioned by participants, 60.2% (*n* = 177) (Table 2). Among 294 participants who were capable of mentioning at least one contraceptive method, most of them 35.5% (*n* = 150) mentioned health facilities as the source of their information (Fig. 2).

**Table 2:** Common contraceptive methods mentioned by participants. (*n* = 294)

Contraceptive method	Frequency (n)	Percent (%)
Condom	177	60.2%
Pills	151	51.4%
Injection	102	34.8%
Intrauterine method	89	30.2%
Implant	83	28.2%
Sterilization method	55	18.7%
Calendar method	40	13.6%
Withdrawal method	44	15.0%
Abstinence	25	8.5%
Emergency contraceptive	4	1.4%

*Abbreviations*: *n* Number of participants, *%* percent of participants

### Association between socio-demographic characteristics and knowledge of contraceptives

Older students aged 17-19 years had more adequate knowledge of contraceptives compared to those aged 13-16 years with the *p-value* of < 0.01 (50.6% vs 28.2% respectively). Being in the lower class was significantly associated with inadequate knowledge of contraceptives. Students from Form I had inadequate knowledge of contraceptives compared to those from Form VI (*p*< 0.01). Students from government schools had more knowledge of contraceptives, 45.7% than students from private schools, 27.2% (*p*=0.01). The relationship was significantly associated with adequate knowledge of contraceptives (*p*< 0.016). Adequate knowledge of contraceptives was higher among students who were not staying with their parents, 62.9% than those students who stayed with both parents 39.9%, with a *p*-value of 0.01 (Table 3).

**Table 3:** Association between socio-demographic characteristics and contraceptive knowledge using a Chi-Square analysis (*n* = 422)

**Variable**	**Contraceptive Knowledge**		***P*- value**
	**Adequate n (%)**	**Inadequate n (%)**	
**Age Group**			
13–16	50 (28.2)	127 (71.8)	** < 0.01**
17–19	124 (50.6)	121(49.4)	
**Age at first sex(*n* = 164)**			
10–13	16 (35.6)	29 (64.4)	0.07I
14–16	40 (44.4)	50 (55.6)	
17–19	20 (69.0)	9 (31.0)	
**Study Year**			
Form I	15 (21.4)	55 (78.6)	
Form II	21 (30.0)	49 (70.0)	
Form III	28 (40.0)	42 (60.0)	** < 0.01**
Form IV	32 (45.0)	39 (55.0)	
Form V	35 (50.0)	35 (50.0)	
Form VI	43 (60.6)	28 (39.4)	
**Religion**			
Muslim	94 (44.3)	118 (55.7)	0.31
Christian	80 (38.1)	130 (61.9)	
**Type of School**			
Government School	147 (45.7)	175 (54.3)	**0.01**
Private School	27 (27.0)	73 (73.0)	
**Number of Family members**			
1–3	10 (34.5)	19 (65.5)	
4–6	109 (41.4)	154 (58.6)	0.25
7 and above	55 (42.3)	75 (57.7)	
**Are you in a relationship?**			
Yes	31 (58.5)	22 (41.5)	**0.02**
No	143 (38.8)	226 (61.2)	
**Stay with parents**			
Yes with Both parents	108 (39.9)	163(60.1)	
Yes with Single parent	44 (37.9)	72 (62.1)	**0.01**
No	22 (62.9)	13 (37.1)	

*Abbreviations*: *n* number participants, *%* percent of participants, *p* Statistical significance at < 0.05 level

### Use of contraceptives among participants

Male students reported that they had sex in their lifetime were 164 out of 422 (38.9%), and the lowest age for first sex was 10 years old. Among students who ever had sex in their lifetime (*N* = 164), only a few of them 29.8% (*n* = 49) reported using any form of contraceptive method, and most of them 69.4% (*n* = 34) used the method to prevent only pregnancy. The main source of contraceptive services was the pharmacy/Chemist shop 55.1% (*n* = 27). Male condoms were the most common contraceptive method used, 69.4%( *n* = 34). The chief reason for reported not using any form of contraceptive was not bothered though sexually active (Table 4).

**Table 4:** Use of contraceptives among respondents

Variables	Frequency (n)	Percent (%)
**Ever had sex (*n* = 422)**		
Yes	164	38.9
No	258	61.1
**Ever use a contraceptive method in your lifetime (*n* = 164)**		
Yes	49	29.9
No	115	70.1
**Reason for not using a contraceptive method (*n* = 115)**		
Not bothered though sexually active	81	70.4
Had side effects	34	29.6
**Participants ever use contraceptive methods (*n* = 49)**		
Reason for using a contraceptive method		
To prevent only pregnancy	34	69.4
To prevent only STDs	10	20.4
To space childbirth	5	10.2
**The place you get contraceptive service (*n* = 49)**		
Hospital	10	20.4
Health Centre	3	6.1
Dispensary	4	8.2
Pharmacy	27	55.1
Others	5	10.2
**Contraceptive method used (*n* = 49)**		
Pills	6	12.2
Male condoms	34	69.4
Female condoms	2	4.1
Injection	2	4.1
Withdrawal method	4	8.2
Calendar method	1	2

*Abbreviations*: *n* Number of participants, *%* percent of participants

### Association between socio-demographic characteristics, contraceptive knowledge, and contraceptive use (*N* = 164)

Age, type of school, relationship status, and knowledge of contraceptives were found to be significantly associated with the use of contraceptives with *p* = 0.008, *p* = 0.046, *p* = 0.002, and *p* = 0.005 consecutively as illustrated in Table 5. The multivariate logistic regression model analysis for the factors significantly associated with the use of contraceptive methods revealed that students who had adequate knowledge of contraceptives were 2 times more likely to use contraceptives compared to those who had inadequate knowledge (AOR = 2.704, 95% CI: 1.220–5.995, *p* = 0.014). The odds of use of contraceptives were increased among students aged 17–19 years although the association was not statistically significant. Students who were aged 17–19 years were 2.2 times more likely to use contraceptives than those aged 13–16 years (AOR = 2.299, 95% CI: 0.805–6.566, *p* = 0.120). Studying in private schools was associated with 4.3 times more likely to use contraceptives than studying in government schools (AOR = 4.347, 95% CI: 1.758–10.762, *p* = 0.01), and students who were in a relationship were 3.5 times than those not in a relationship to use of the contraceptive method (AOR = 3.51, 95% CI: 1.421–8.670, *p* = 0.006) (Table 6).

**Table 5:** Association between socio-demographic characteristics, knowledge of contraceptives, and contraceptive use among participants who ever had sex using a Chi-Square analysis (*N* = 164)

**Variable**	**Ever use a contraceptive method**		
	**Yes. N (%)**	**No N (%)**	***P*-value**
**Contraceptive Knowledge**			
Adequate 31(40.8)		45(59.2)	**0.005**
Inadequate 18 (38.3)		70(61.7)	
**Age Group**			
13–16	6(14.0)	37(86.0)	**0.008**
17–19	43(35.5)	78(64.5)	
**Age at first sex**			
10–13	13(28.9)	32(71.1)	0.98
14–16	27(30.0)	63(70.0)	
17–19	9(31.0)	20(69.0)	
**Class**			
Form I	4(16.7)	20(83.3)	
Form II	3(13.6)	19(86.4)	
Form III	6(30.0)	14(70.0)	0.199
Form IV	11(37.9)	18(62.1)	
Form V	12(33.3)	24(66.7)	
Form VI	13(39.4)	20(60.6)	
**Religion**			
Muslim	22(27.2)	59(72.8)	0.453
Christian	27(32.5)	56(67.5)	
**Type of School**			
Government School	31(25.6)	90(74.4)	**0.046**
Private School	18(41.9)	25(58.1)	
**Number of Family members**			
1–3	0(0.0)	9(100)	
4–6	37(34.6)	70(65.4)	0.064
7 and above	12(25.0)	36(75.0)	
**Are you in a relationship?**			
Yes	18(51.4)	17(48.6)	**0.002**
No	31(24.0)	98(76.0)	
**Stay with parents**			
Yes with Both parents	34(30.1)	79(69.9)	
Yes with Single parent	13(33.3)	26(66.7)	0.542
No	2(16.7)	10(83.3)	

*Abbreviations*: *N* Number of participants, *%* percent of participants, *p* Statistical

**Table 6:** Multivariate logistic regression analysis of the predictors of contraceptive use among participants who ever had sex (*N* = 164)

Variables	*p*-value	COR	95% C.I	*p*-value	AOR	95% C.I
**Contraceptive knowledge**						
Inadequate	**-**	1	-	-	1	-
Adequate	**0.005**	2.679	1.342–5.347	**0.014**	2.704	1.220–5.995
**Age group**						
13–16	**-**	1	-	-	1	-
17–19	**0.011**	3.400	1.329–8.698	**0.12**	2.299	0.805–6.566
**Types of School**						
Government School	**-**	1	-	-	1	-
Private School	**0.048**	2.09	1.007–4.340	**0.01**	4.347	1.758–10.762
**Relationship status**						
No	**-**	1	-	-	1	-
Yes	**0.002**	3.347	1.540–7.274	**0.006**	3.51	1.421–8.670

*Abbreviations*: *COR* Crude Odds ratio, *AOR* Adjusted Odds Ratio, *95% CI* 95% confidence interval, *p* Statistical significance at < 0.05 level, *1* Category reference

## Discussion

This study examined the factors associated with the uptake of contraceptives among male secondary school adolescents in Kisarawe District, Coast Region-Tanzania. It found that the majority of male adolescents had inadequate knowledge of contraceptives although most of them were able to mention at least one contraceptive method which was similar to a study conducted in Kintampo, Ghana, where 92.1% of male adolescents were able to mention at least one contraceptive method [25]. This finding is contentious from the survey conducted in Hai District-Tanzania, whereby a majority of male adolescents had adequate knowledge of contraceptives [23]. This could be due to the cultural background of parents from northern Tanzania to communicate with their children about the importance of education and the strategies to cope with sexual behaviors. The results of the study conducted in Kenya mentioned Health facilities as the major source of contraceptive information among adolescents which correlated with the current study [26], This might be due to the hardship of accessing other sources of contraceptive information such as Television/Radio or Phones, and parents in rural areas.

Contraceptive knowledge by method was higher on male condoms compared to other contraceptive methods. This was similar to the study findings conducted in several areas in Africa [10, 27, 28], and the reason for this high awareness of condoms might be due to the HIV/AIDS prevention campaign. Almost all media around the world advertise on use of condoms for dual protection of HIV and pregnancy which might contribute to high awareness of condoms.

The older students had higher knowledge of contraceptives compared to younger ones, which was not the same as the study conducted in Semi-urban settlements in Nigeria [29]. Students from lower classes displayed lower knowledge of contraceptives compared to those in higher classes, which was similar to the survey conducted in the Hai district, Tanzania [23], and this could be because of Tanzania secondary school curriculum where the sexual and reproductive course start to be taught from students of year 3 (form 3) [30]. Those students who were in a relationship had more knowledge of contraceptives than those who were not in a relationship which corresponds to a survey conducted in some selected African countries, and this might be due to sexual experience and more likely to seek contraceptive information among students who were in a relationship [31]. Students who were studying in government schools had a higher contraceptive knowledge than those students from private schools, and this difference could be due to the sample size where a majority of students were from government schools. Male adolescents who were living with their parents had low contraceptive knowledge compared to those who were not living with their parents, this might be due to parents perceiving that discussing sexual matters with their children is a taboo [33]. In Tanzania, efforts have been made to increase convenient access to parent-adolescent communication on Sexual and Reproductive issues to empower adolescents to make responsible decisions regarding sexual manners [34]. Unfortunately despite all that the practice is still low, and the study conducted in the southern and central parts of Tanzania showed that girls are more likely to communicate Sexual and Reproductive information with their parents than boys [35]. A study conducted at Free State School of Nursing, South Africa discovered poor communication on sexual issues and misleading information on contraceptive use between father and their boys in the community contribute to unsafe sexual activity and increase the rate of unintended teenage pregnancy [36]**.**

Also, this study found that the rate of contraceptive use among male adolescents who ever had sex to be low (29.9%), this corresponded with many studies conducted among male adolescents [10, 23, 25, 37]. The lowest age at first sex was 10 years old which was different compared to the data obtained in 2016 from Tanzania Demographic and Health Survey and Malaria Indicator Survey in which the lowest age at the first sex among adolescents was 12 years old [21]. This finding indicates the need to inform adolescents about contraceptive information at a very early age. The major source of the contraceptive method was pharmacy/chemist shops. This might be explained due to the easiness of students to access pharmacy/ chemist shops, and the environment is youth-friendly compared to other sources of contraceptive services. A male condom was the most common contraceptive method used. The finding has been common in various studies conducted among adolescents exploring their contraceptive use [10, 11, 21, 38] The fact that it’s very easy to access male condoms unlikely to other contraceptive methods. A male condom can be found everywhere unlike other methods that require adolescents to get the service from health facilities. Most of the health facilities are not youth-friendly limiting them to seek services from other methods of contraceptive [25]. Tanzania through the Ministry of Health established quality Adolescent-Friendly Health Service Centres as a strategy to support and protect the health of adolescents [39]. However, the project has not adopted fully as many used facility-based models that allow the utilization of already existing resources to provide services to adolescents [40]. In 2019, only 30% of health services met national standards for Adolescent-Friendly Health, and the speed of training youth-friendly service providers in Tanzania has been slow [41, 42]. In total about 78% of facilities had separate waiting and counseling rooms for adolescents, and in the Coastal region 57% of facilities had separate palaces for adolescents [39].

The age, type of school, relationship status, and contraceptive knowledge were found to be the predictors of the use of contraceptives among male adolescents. Older adolescents reported using contraceptive methods more than early adolescents. A similar result was revealed in studies conducted in Burkina Faso, Nigeria, and Ethiopia [43]. Students studying in private schools increased the trend of using contraceptives among adolescents compared to those studying in government schools. Being in a relationship increased the trend of contraceptive use more than not being in a relationship among adolescents, this is similar to a study conducted in Santiago Island-Cape Verde [44]. Having adequate knowledge of contraceptives increased the trend of using contraceptive methods among male adolescents. Contraceptive knowledge has been recommended by many researchers as the greatest approach to increase the rate of contraceptive use among adolescents as it decreases misconceptions, and supports decision-making and communication skills among adolescents toward contraceptive use [45].

Furthermore, although most adolescents were able to mention at least one contraceptive method, that does not translate into use. This could be due to male adolescents' lack of deep and complete knowledge of effectiveness, side effects, misconceptions, sources, practice, and access to various types of contraceptive methods. A study conducted in Nigeria showed that adolescents were not utilizing contraceptives because they lacked enough knowledge of contraceptive services, and they were afraid to suffer from the side effects of using contraceptives. Consequently, lose a chance of having a baby in the future [29].

## Limitations of the study

This study addressed a very sensitive subject in which male adolescents were asked private questions regarding their sexual issues. This kind of question has a high possibility of creating an uncomfortable situation for adolescents to give out their true response which could disturb the reliability and validity of the data. Confidentiality and working trust with adolescents and some of the teachers were established during the process of data collection to ensure adolescents were easy to give out the response and minimize the effect. A well-trained research assistant was present in the classroom to reply to possible questions and the questionnaire was simplified for easy understanding.

## Conclusion

This study found low use of contraceptives among male adolescents despite most of them being able to mention at least one contraceptive method. This finding provides very useful pieces of information to policymakers and other stakeholders working on increasing the use of contraceptives and reducing teenage pregnancy. It’s paramount to integrate Comprehensive Sexuality Education to be taught from a very young age as currently, adolescents initiate sex at an early age and to be tailored to involve males as the key players. If possible this education to be taught by young well-skilled professionals rather than old people, this can help to raise the confidence and cooperation of adolescents during the intervention.

## Data Availability

When necessary, the research tools, dataset, and other materials supporting the results will be shared upon consultation with the corresponding author.
